# Effects of Antipsychotic Drugs on the Epigenetic Modification of Brain-Derived Neurotrophic Factor Gene Expression in the Hippocampi of Chronic Restraint Stress Rats

**DOI:** 10.1155/2018/2682037

**Published:** 2018-06-11

**Authors:** Mi Kyoung Seo, Young Hoon Kim, Roger S. McIntyre, Rodrigo B. Mansur, Yena Lee, Nicole E. Carmona, Ah Jeong Choi, Gyung-Mee Kim, Jung Goo Lee, Sung Woo Park

**Affiliations:** ^1^Paik Institute for Clinical Research, Inje University, Busan, Republic of Korea; ^2^Department of Psychiatry, Gongju National Hospital, Gongju, Republic of Korea; ^3^Mood Disorders Psychopharmacology Unit, University Health Network, University of Toronto, Toronto, ON, Canada; ^4^Department of Psychiatry, University of Toronto, Toronto, ON, Canada; ^5^Department of Psychiatry, College of Medicine, Haeundae Paik Hospital, Inje University, Busan, Republic of Korea; ^6^Department of Health Science and Technology, Graduate School, Inje University, Busan, Republic of Korea; ^7^Department of Convergence Biomedical Science, College of Medicine, Inje University, Busan, Republic of Korea

## Abstract

Recent studies have shown that antipsychotic drugs have epigenetic effects. However, the effects of antipsychotic drugs on histone modification remain unclear. Therefore, we investigated the effects of antipsychotic drugs on the epigenetic modification of the BDNF gene in the rat hippocampus. Rats were subjected to chronic restraint stress (6 h/d for 21 d) and then were administered with either olanzapine (2 mg/kg) or haloperidol (1 mg/kg). The levels of histone H3 acetylation and MeCP2 binding at BDNF promoter IV were assessed with chromatin immunoprecipitation assays. The mRNA levels of total BDNF with exon IV, HDAC5, DNMT1, and DNMT3a were assessed with a quantitative RT-PCR procedure. Chronic restraint stress resulted in the downregulation of total and exon IV BDNF mRNA levels and a decrease in histone H3 acetylation and an increase in MeCP2 binding at BDNF promoter IV. Furthermore, there were robust increases in the expression of HDAC5 and DNMTs. Olanzapine administration largely prevented these changes. The administration of haloperidol had no effect. These findings suggest that the antipsychotic drug olanzapine induced histone modification of BDNF gene expression in the hippocampus and that these epigenetic alterations may represent one of the mechanisms underlying the actions of antipsychotic drugs.

## 1. Introduction

Schizophrenia is a complex and chronic mental illness with a lifetime prevalence of approximately 1% [1, 2]. It is a genetic disorder with a heritability of up to 80% [3], and the majority of patients with schizophrenia suffer from psychotic, affective, and cognitive symptoms as well as functional impairments [4–6]. Although advances have been made in the treatment of schizophrenia, its etiology and pathophysiology have yet to be fully elucidated. In fact, heterogeneous phenotypes and lack of clear pathological lesions in the brain have been major hurdles in the field of schizophrenia research [7].

Of the suggested models that describe the development of schizophrenia, neurodevelopmental and neurodegeneration models represent two nonmutually exclusive theories regarding the etiology and course of this disorder [7–10]. Furthermore, the roles of multiple epigenetic mechanisms in the development of schizophrenia are now becoming a primary focus of research [7, 10, 11]. Epigenetic modifications are most commonly regulated by DNA methylation and histone modification [12–14]. The entire length of DNA in a single somatic cell exists within the nucleus complex along with chromatin [15, 16]. The primary structural unit of chromatin is the nucleosome, which is composed of the standard length of DNA and four pairs of basic histone proteins (H2A, H2B, H3, and H4) [17, 18]. In vertebrates, methylation of the CpG dinucleotide within proximal promoters is frequently linked to transcriptional repression (i.e., gene silencing) [19]. The gene silencing effect of DNA methylation is mediated by methyl-CpG-binding protein 2 (MeCP2), which is one of several CpG-binding proteins [20, 21], which recruits histone deacetylases (HDACs) to remove active modifications and repress gene transcription [20, 21]. MeCP2 can also enhance the repressive chromatin state via the addition of repressive H3K9 methylation to histone methyltransferase [19, 22].

An increasing amount of evidence suggests that epigenetic modifications in certain brain regions and neural circuits may be important mechanisms underlying the development of schizophrenia [23–26]. The altered functioning of cortical pyramidal neurons and cortical parvalbumin- (PV-) positive GABAergic interneurons may be related to the psychotic and cognitive symptoms of schizophrenia [27–29]. Huang and Akbarian found an average 8-fold deficit in repressive chromatin-associated DNA methylation at GAD1 promoters in patients with schizophrenia [30] and it has been shown that histone 3 (H3), one of the histone proteins, was particularly dysregulated in schizophrenia patients [31]. Furthermore, the di- and trimethylation of H3K9 and H3K17, which are known to regulate GAD1 expression, were found to be elevated in cortical neurons in a postmortem study of patients with schizophrenia [31].

Following the introduction of chlorpromazine in 1952, antipsychotic drugs have been widely used for the treatment of schizophrenia [1, 32]. Subsequently, as research data have accumulated, it has been suggested that antipsychotic drugs may be involved in the regulation of epigenetic changes in the brain. An in vivo study by Dong et al. [33] demonstrated that the antipsychotic drugs clozapine and sulpiride increased the cortical and striatal demethylation of hypermethylated RELN and GAD1 promoters in the prefrontal cortex of mice after 7 d of methionine treatment. Clozapine attenuated the decrease in histone H3 acetylation at lysine 9 residues in the prefrontal cortex and ameliorated memory impairments and social deficits in mice treated with phencyclidine [34]. Additionally, Melka et al. [35] reported that olanzapine altered methylation in genes associated with dopamine neurotransmission in the hippocampus and cerebellum of rats. In humans, frontocortical DNA methylation of the BDNF gene was correlated with a genotype that was associated with major psychosis in a genome-wide epigenomic study of major psychosis [36]. Moreover, Abdolmaleky et al. [37] showed that antipsychotic drugs attenuated the aberrant DNA methylation of the dysbindin (BTNBP1) promoter in the saliva and postmortem brain samples of patients with schizophrenia and psychotic bipolar disorder.

BDNF is a neurotrophic factor and, as mentioned above, epigenetic changes of the BDNF gene are related to the pathophysiology of schizophrenia [7, 38, 39]. The reduced expression of BDNF and increases in the promoter methylation of BDNF exons IV and IX have been identified in the frontal cortex and hippocampus of patients with schizophrenia [40, 41]. Although it may be postulated that antipsychotic drugs influence the epigenetic mechanisms associated with the BDNF gene, there is a relative lack of studies investigating the epigenetic effects of antipsychotic drugs on the BDNF gene in the brain. Therefore, the present study evaluated the manner in which antipsychotic drugs alter epigenetic changes in the BDNF gene in the rat hippocampus. To assess this, we used a chronic restraint stress model to induce morphological and functional changes in the hippocampus [42–44]. A recent study from our research group demonstrated that chronic restraint stress decreased the levels of BDNF expression and acetylated histone H3 at BDNF promoter IV in the rat hippocampus [45]. Epigenetic changes in the BDNF gene induced by antipsychotic drugs may be more apparent when measured under conditions of reduced BDNF or acetylated histone levels. Furthermore, certain atypical antipsychotic drugs, but not typical antipsychotic drug haloperidol, attenuated chronic restraint stress-induced decreases in BDNF expression level [46–48]. As a result, the primary aims of the present study were to investigate the levels of total and exon IV BDNF mRNA and the levels of histone H3 acetylation and MeCP2 binding at BDNF promoter IV. Additionally, the mRNA levels of HDAC5, DNMT1, and DNMT3a in the hippocampus were assessed following the chronic administration of olanzapine and haloperidol to rats that were either exposed or not exposed to 21 d of chronic restraint stress.

## 2. Materials and Methods

### 2.1. Animals and Drug Administration

All experiments involving animals were approved by the Committee for Animal Experimentation and the Institutional Animal Laboratory Review Board of Inje Medical College (approval number 2015-029). For the present study, male Sprague-Dawley rats (Orient Bio, Gyeonggi-Do, Korea) weighing 200–250 g were housed 2 or 3 per cage with ad libitum food and water and maintained at 21°C on a 12/12 h light/dark cycle. After 7 d of acclimatization, the rats were randomly divided into 6 groups of 6 rats each (*n* = 6 animals/group). All drugs were dissolved in vehicle (0.8% glacial acetic acid in 0.9% saline) and injected intraperitoneally (i.p.) into the animals. The first group (vehicle control) received vehicle (1 mL/kg, i.p.) without restraint stress; the second (olanzapine) and third (haloperidol) groups received olanzapine (2 mg/kg, i.p.) and haloperidol (1 mg/kg, i.p.), respectively, without restraint stress; the fourth group (vehicle + stress) received vehicle at 10:00 and was then completely restrained for 6 h from 11:00 to 17:00 in specially designed plastic restraint tubes (dimensions: 20 cm high, 7 cm in diameter); and the fifth (olanzapine + stress) and sixth (haloperidol + stress) groups received olanzapine (2 mg/kg, i.p.) and haloperidol (1 mg/kg, i.p.), respectively, and were then immobilized in the same way as the rats in the fourth group. All procedures were repeated once daily for 3 weeks. In our preliminary experiment, the expression patterns of BDNF according to time (2 and 6 h) and period (1, 7, and 21 d) of restraint stress were analyzed by Western blotting (Supplementary Materials; Figure S1). Six-hour-daily stress for 1, 7, and 21 d significantly reduced BDNF expression levels. To assess the chronic effects of antipsychotic drugs on restraint stress, a stress period of 21 d was used.

Olanzapine was supplied by Eli Lilly Research Laboratories (Indianapolis, IN, USA), and haloperidol was purchased from Sigma (St. Louis, MO, USA). The clinical effects of many antipsychotic drugs are reflected by a dopamine D2 receptor occupancy of 60–70% [49, 50]. All drug doses in the present study were calculated based on rat studies that investigated D2 receptor occupancy [51, 52] and produced plasma levels well within the therapeutic range of the doses used for the clinical treatment of patients with schizophrenia [53].

### 2.2. Measurement of mRNA Levels by Quantitative Real-Time Polymerase Chain Reaction (qRT-PCR)

Total RNA was isolated using TRIzol® (Invitrogen, Carlsbad, CA, USA) as previously described by Seo et al. [45]. RNA samples were reverse transcribed into cDNA using amfiRivertII™ cDNA Synthesis Master Mix (GenDepot, Baker, TX, USA), and a qRT-PCR procedure was performed using SYBR® Green Supermix (Bio-Rad, Hercules, CA, USA) and the CFX96™ Real-Time PCR Detection System (Bio-Rad). The specificity of amplification was verified by melting curves. The oligonucleotide sequences of the primers used in the present study were described previously [45], and the qRT-PCR procedure for DNMT1 and DNMT3a was performed with the following primers: DNMT1, forward 5′-GAGTGGGA TGGCTTCTTCAG-3′, reverse 5′-GTGTCTGTCCAGGATGTTG C-3′; DNMT3a, forward 5′-ACGCCAAAGAAGTGTCTGCT-3′, reverse 5′-CTTTGCCCTG CTTTATGCAG-3′; and glyceraldehyde 3-phosphate dehydrogenase (GAPDH), forward 5′-TCCCTCAAGATTGTCAGCAA-3′, reverse 5′-AGATCCACAACGGA TACATT-3′. ΔCt, which represents the difference between GAPDH and the target gene samples, was calculated using the following formula: ΔCt = Ct_target gene_ − Ct_GAPDH_. Then, the fold difference was quantified using the 2^−△△ct^ method; the final value was expressed as a value relative to the vehicle control. All samples were assayed in twice.

### 2.3. Chromatin Immunoprecipitation (ChIP) Assays

The ChIP assays in the present study were performed as described previously [45]. Hippocampal samples were cross-linked, homogenized, and sonicated to generate 200–500 bp chromatin fragments. Then, chromatin lysate (10 *μ*g) was immunoprecipitated with an antibody (10 *μ*g) directed against either acetyl-histone H3 (K9 + K14; 06–599; Millipore, Billerica, MA, USA) or MeCP2 (ab2828; Abcam, Cambridge, UK). Protein-associated chromatin was extracted in phenol/chloroform (Amresco, Solon, OH, USA) and precipitated in ethanol (Merck, Hunterdon, NJ, USA) prior to qRT-PCR analysis. The ChIP results were normalized to the input DNA. ΔCt, which represents the difference between the input and immunoprecipitated samples, was calculated using the following formula: ΔCt = Ct_ip_ − Ct_input_. Then, the fold difference was quantified using the 2^−△△ct^ method, and the final value was expressed as a value relative to the vehicle control. All samples were assayed in twice.

### 2.4. Statistical Analysis

All statistical analyses were performed using GraphPad Prism version 7.01 (GraphPad Software, La Jolla, CA, USA). A two-way analysis of variance (ANOVA) was performed to determine whether the main effects of restraint stress and treatment and the interactive effect of restraint stress × treatment were significant. Tukey's multiple comparison tests were used for post hoc comparisons. *p* values ≤ 0.05 were considered to indicate statistical significance. All data are presented as a mean ± standard error of the mean (SEM).

## 3. Results

Individual-level data for each group analyzed by qRT-PCR and ChIP are shown in Table 1.

### 3.1. Effects on Hippocampal BDNF mRNA Levels

**Figure 1:**
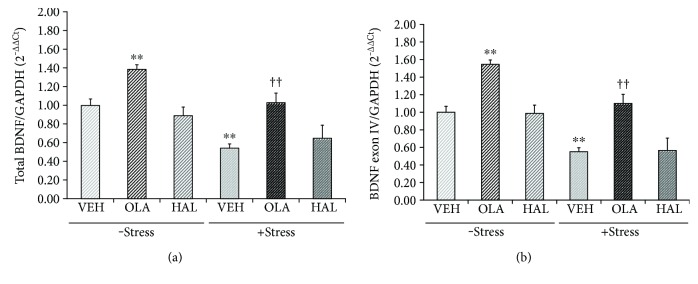
Effects of antipsychotic drugs on total and exon IV brain-derived neurotrophic factor (BDNF) mRNA levels in the rat hippocampus. Rats were given a daily injection of either vehicle (VEH), olanzapine (OLA), or haloperidol (HAL) for 21 d in conjunction with either exposure to (+ stress) or no exposure to (− stress) restraint stress. The mRNA levels of total BDNF (a) and exon IV BDNF (b) in the rat hippocampus were measured using a quantitative real-time polymerase chain reaction (qRT-PCR) procedure. The quantitative analysis was normalized to glyceraldehyde-3-phosphate dehydrogenase (GAPDH). ^∗∗^ *p* < 0.01 versus vehicle control; ^††^ *p* < 0.01 versus vehicle + stress.

A two-way ANOVA assessing total BDNF mRNA levels (Figure 1(a)) revealed significant main effects of restraint stress (*F*_[1,30]_ = 66.370, *p* < 0.001) and treatment (*F*_[2,30]_ = 38.500, *p* < 0.001) and a significant interaction effect of restraint stress × treatment (*F*_[2,30]_ = 4.043, *p* = 0.028). A post hoc analysis for the main effect of restraint stress revealed that stress decreased the level of total BDNF mRNA relative to the vehicle control (*p* = 0.001), while the post hoc analysis for the main effect of treatment revealed that chronic olanzapine, but not chronic haloperidol, administration increased the expression of total BDNF mRNA compared with the vehicle control group in the stress-free condition (*p* = 0.009) and reversed the stress-induced decrease in BDNF levels (*p* < 0.001). The significant interaction effect of stress × treatment indicated that the positive effect of olanzapine on the level of BDNF expression was greater in the stress condition than in the stress-free condition.

The present study also investigated whether antipsychotic drugs influenced exon IV BDNF mRNA expression in the stress-free and stress conditions (Figure 1(b)). There were significant main effects of restraint stress (*F*_[1,30]_ = 120.600, *p* < 0.001) and treatment (*F*_[2,30]_ = 67.790, *p* < 0.001) and a trend for an interaction effect of restraint stress × treatment (*F*_[2,30]_ = 3.091, *p* = 0.060). The post hoc analyses for the main effects of restraint stress and treatment revealed that chronic stress significantly reduced the levels of exon IV BDNF mRNA compared with the vehicle control group (*p* < 0.001). This reduction was reversed by chronic olanzapine, but not chronic haloperidol, treatment (*p* < 0.001). Additionally, chronic olanzapine, but not chronic haloperidol, treatment increased exon IV mRNA levels in the stress-free condition (*p* < 0.001).

### 3.2. Effects on the BDNF Promoter IV Epigenetic State in the Hippocampus

**Figure 2:**
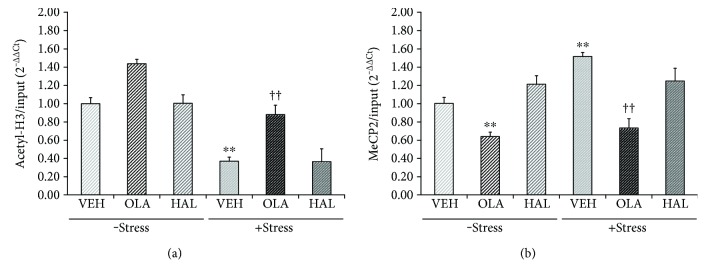
Effects of antipsychotic drugs on acetylated histone H3 and methyl CpG-binding protein 2 (MeCP2) levels at BDNF promoter IV in the rat hippocampus. Rats were given a daily injection of either VEH, OLA, or HAL for 21 d in conjunction with either + stress or − stress. Chromatin immunoprecipitation (ChIP) assays were performed to measure the levels of acetylated H3 (a) and MeCP2 (b) at BDNF promoter IV in the rat hippocampus using specific antibodies. These levels were quantified by a qRT-PCR procedure. ^∗∗^ *p* < 0.01 versus vehicle control; ^††^ *p* < 0.01 versus vehicle + stress.

To determine whether the antipsychotic drugs affected histone modification at promoter IV of the BDNF gene, ChIP assays were performed to evaluate the levels of acetylated histones H3 and MeCP2 binding in BDNF promoter IV. When the levels of histone H3 acetylation (Figure 2(a)), a marker of transcriptional activation, were examined following chronic treatment with olanzapine and haloperidol in the stress-free and stress conditions, significant main effects of restraint stress (*F*_[1,30]_ = 124.500, *p* < 0.001) and treatment (*F*_[2,30]_ = 30.500, *p* < 0.001) and a significant interaction effect of restraint stress × treatment (*F*_[2,30]_ = 5.363, *p* = 0.010) were detected. Post hoc analyses revealed that stress significantly reduced the level of acetylated histone H3 (*p* < 0.001) and that chronic treatment with olanzapine reversed this reduction (*p* < 0.001). Olanzapine treatment did not significantly alter the level of acetylated histone H3 in the stress-free condition (*p* = 0.086), although it appeared to increase histone H3 levels. In contrast, chronic haloperidol treatment did not affect histone H3 levels irrespective of exposure to restraint stress.

MeCP2 binds to the cyclic adenosine monophosphate (AMP) response element (CRE) site within BDNF promoter IV [54]. MeCP2 can decrease BDNF expression and suppress the transcription of promoter IV by blocking the binding of CRE-binding protein (CREB), a transcription factor, to CRE [54, 55]. A two-way ANOVA revealed significant main effects of restraint stress (*F*_[1,30]_ = 9.214, *p* = 0.005) and treatment (*F*_[2,30]_ = 38.120, *p* < 0.001) and a significant interaction effect of restraint stress × treatment (*F*_[2,33]_ = 3.280, *p* = 0.050). The level of MeCP2 binding at promoter IV (Figure 2(b)) increased after chronic stress (*p* = 0.009), but this increase was reversed by chronic olanzapine (*p* < 0.001), but not chronic haloperidol, treatment. Additionally, treatment with olanzapine, but not haloperidol, decreased MeCP2 levels in the stress-free condition (*p* = 0.004) and this reduction was greater in the stress condition (2.07-fold decrease) than in the stress-free condition (1.56-fold decrease).

### 3.3. Effects on HDAC5 mRNA Level in the Hippocampus

**Figure 3:**
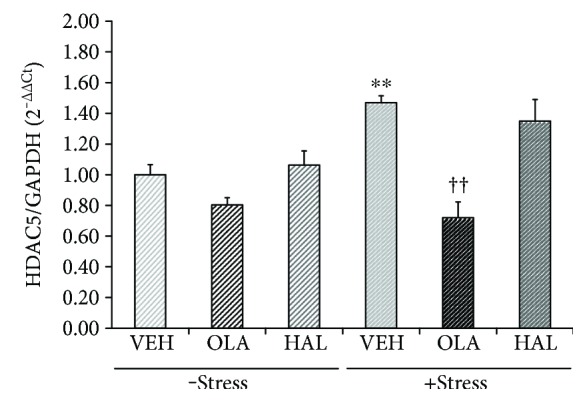
Effects of antipsychotic drugs on histone deacetylase 5 (HDAC5) mRNA levels in the rat hippocampus. Rats were given a daily injection of either VEH, OLA, or HAL for 21 d in conjunction with either + stress or − stress. HDAC5 mRNA levels in the rat hippocampus were assessed using a qRT-PCR procedure. The quantitative analysis was normalized to GAPDH. ^∗∗^ *p* < 0.01 versus vehicle control; ^††^ *p* < 0.01 versus vehicle + stress.

HDACs can participate in gene silencing due to their binding to MeCP2 along with other corepressors, such as mSin3A [20, 21, 55]. In particular, mechanistic evidence for the role of HDAC5, which is a class II HDAC, was identified in rodents that administered with antidepressant drugs and exposed to stressful environments [45, 56–58]. To investigate the mechanisms underlying histone modification induced by restraint stress and olanzapine treatment observed in the present study, HDAC5 expression levels were measured using qRT-PCR (Figure 3).

There were significant main effects of restraint stress (*F*_[1,30]_ = 10.550, *p* = 0.003) and treatment (*F*_[2,30]_ = 34.110, *p* < 0.001) on the HDAC5 expression level as well as a significant interaction effect of restraint stress × treatment (*F*_[2,30]_ = 7.788, *p* = 0.002). A post hoc analysis of all groups revealed that HDAC5 levels increased only in the restraint stress group (*p* = 0.003) and that this increase was blocked by olanzapine treatment (*p* < 0.001). In contrast, chronic treatment with haloperidol had no effect on the HDAC5 level in the hippocampi of rats in either the stress-free or stress condition.

### 3.4. Effects on DNMT1 and DNMT3a mRNA Levels in the Hippocampus

**Figure 4:**
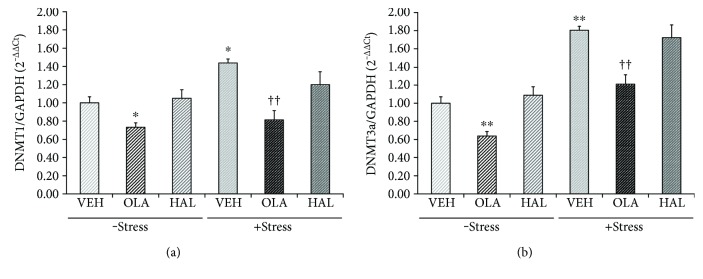
Effects of antipsychotic drugs on DNA methyltransferase (DNMT) 1 and DNMT3a mRNA levels in the rat hippocampus. Rats were given a daily injection of either VEH, OLA, or HAL for 21 d in conjunction with either + stress or − stress. The DNMT1 (a) and DNMT3a (b) mRNA levels in the rat hippocampus were assessed using a qRT-PCR procedure. The quantitative analysis was normalized to GAPDH. ^∗^ *p* < 0.05 versus vehicle control, ^∗∗^ *p* < 0.01 versus vehicle control, and ^††^ *p* < 0.01 versus vehicle + stress.

DNA methylation is dependent on the enzymatic function of several DNA methyltransferases, including DNMT1, DNMT3a, and DNMT3b [59]. In particular, DNMT1 and DNMT3a are required for maintaining DNA methylation in central nervous system neurons in adult mice [60]. Therefore, the influence of antipsychotic drugs on DNMT1 (Figure 4(a)) and DNMT3a (Figure 4(b)) mRNA levels in the stress-free and stress conditions was investigated in the present study.

A two-way ANOVA revealed significant main effects of restraint stress (DNMT1: *F*_[1,30]_ = 12.380, *p* = 0.001 and DNMT3a: *F*_(1,30)_ = 115.900, *p* < 0.001) and treatment (DNMT1: *F*_[2,30]_ = 23.270, *p* < 0.001 and DNMT3a: *F*_[2,30]_ = 30.460, *p* < 0.001) for these enzymes but not an interaction effect. A post hoc analysis revealed that restraint stress resulted in an overall upregulation of DNMT1 (*p* = 0.010) and DNMT3a (*p* < 0.001) levels; chronic olanzapine, but not chronic haloperidol, treatment induced a downregulation of these levels in both the stress-free (DNMT1: *p* = 0.039 and DNMT3a: *p* < 0.001) and stress (DNMT1: *p* < 0.001 and DNMT3a: *p* = 0.002) conditions.

## 4. Discussion

In the present study, the 21 d restraint stress procedure affected BDNF mRNA expression levels and the epigenetic regulation of BDNF promoter exon IV, HDAC5, DNMT1, and DNMT3a in the rat hippocampus. Chronic restraint stress also decreased the levels of total and exon IV BDNF mRNA, decreased acetylated histone H3 and increased MeCP2 at promoter IV of the BDNF gene, and increased the levels of HDAC5, DNMT1, and DNMT3a mRNA expression. Changes induced by 21 d restraint stress were attenuated by olanzapine administration, while haloperidol did not influence the total and exon IV BDNF levels or BDNF epigenetic regulation.

Similar changes have been reported in previous studies. For example, 21 d restraint stress significantly reduced BDNF expression in the rat hippocampus [61] and decreased BDNF exon IV levels in both the mouse and rat hippocampi [62, 63], which lends support to the present findings. It has also been reported that olanzapine increases total BDNF mRNA levels and normalizes MK-801-induced reductions of BDNF mRNA expression in the rat hippocampus [64, 65]. In the present study, the decreases in total and BDNF exon IV mRNA induced by restraint stress were rescued by treatment with olanzapine; however, the administration of haloperidol failed to show any effects.

The present study also demonstrated that 21 d restraint stress decreased the levels of acetylated histone H3 at promoter IV of the BDNF gene and that this decrease was reversed by olanzapine administration. Several studies have reported interactions between stress and the histone modification of the BDNF gene in the rat and mouse hippocampus. For example, Fuchikami et al. [62] reported that single immobilization stress resulted in a significant decrease in the levels of acetylated histone H3 at promoter IV of the BDNF gene at 2 and 24 h [62], while another study reported that 21 d restraint stress caused mild increases in the levels of H3K4me3 and reductions in H3K9me3 levels in the dentate gyrus [66]. The acetylation of lysine residues at the N-terminus of histone proteins reduces positive net charges which, in turn, results in a reduction in the affinity between histones and DNA. This exposure allows for the binding of transcription factors to the promoters of specific genes. The K9, K14, K18, K23, K27, and K36 regions of histone H3 are acetylated, and acetylation at K9 and K14 of histone H3 enhances transcription [67, 68]. Thus, the present study utilized antibodies that detected histone H3 acetylated at K9 and K14.

MeCP2 is a transcriptional regulator that plays a role in the structural stability of chromatin [69]. Thus, mutations that disrupt MeCP2 function can be expected to increase gene expression, disturb neuronal function, and give rise to behavioral disorders, such as Rett syndrome [69, 70]. In the present study, the administration of olanzapine decreased MeCP2 levels at BDNF promoter IV in rats exposed to 21 d restraint stress, which is similar to previous studies showing increased MeCP2 following exposure to stress. For example, Seo et al. [45] reported that 21 d restraint stress and maternal separation independently increased MeCP2 levels at BDNF promoter IV in the rat hippocampus. However, it has also been shown that prolonged prenatal stress (PNS) did not significantly change MeCP2 levels in either the prefrontal cortex or hippocampus of PNS mouse offspring [71]. The present finding that olanzapine administration decreased MeCP2 occupancy in the BDNF promoter IV region of rats exposed to 21 d restraint stress suggests that olanzapine may regulate MeCP2-dependent transcription of the BDNF gene.

DNA methylation is associated with common and critical processes in mammals, including transposon silencing and genomic imprinting [72]. The DNMT3a, DNMT3b, and DNMTl proteins are primarily responsible for the establishment of genomic DNA methylation patterns and play important roles in human development, reproduction, and mental health [73]. Boersma et al. [74] found that PNS decreased total BDNF expression and increased DNMT1 and DNMT-3a expression in the amygdala and hippocampus of PNS-exposed offspring. Another study showed that clozapine reduced stress-induced elevations in DNMT1 binding to BDNF promoters in the frontal cortex of PNS-exposed offspring [71]. Although the method of stress induction used in the present study differed from those used in these previous studies, 21 d restraint stress also elevated the levels of DNMT1 and DNMT3a mRNA expression in the rat hippocampus; however, these increases were subsequently reversed by olanzapine. In contrast, haloperidol, a typical antipsychotic drug, did not alter these levels.

Histone acetylation and deacetylation remodel chromatin structures and regulate gene expression [75]. The functions of HDACs include the removal of acetyl groups from the N-terminus of the lysine tail; this modification of chromatin results in a compact chromatin structure that prevents an interaction between DNA and regulatory proteins which, in turn, blocks gene expression [76]. Tsankova et al. reported that chronic imipramine treatment reversed this downregulation and increased histone acetylation at BDNF promoters III and VI in the mouse hippocampus [56]. These authors also showed that the hyperacetylation induced by chronic imipramine treatment was associated with the selective downregulation of HDAC5 mRNA levels. Chronic unpredictable stress (CUS) significantly decreased the acetylation rates of H3 at K9 and H4 at K12 and resulted in obvious increases in HDAC expression in the rat hippocampus [57]. However, the administration of sodium valproate, a HDAC5 inhibitor, clearly blunted these decreases in acetylation and blocked the increase in HDAC5 expression [57]. These findings suggest that HDAC5 expression may be involved in the regulation of stress-induced histone acetylation. In the present study, olanzapine blocked the increase in HDAC5 mRNA expression induced by 21 d restraint stress, which indicates that it may affect the epigenetic regulation of HDAC5.

In this study, olanzapine, but not haloperidol, induced epigenetic modifications. Olanzapine is an antipsychotic drug, and the epigenetic modifications that it induces differ from those that may result from haloperidol treatment. It is important to elucidate the molecular mechanisms underlying the epigenetic effects of olanzapine and haloperidol. Although it remains to be confirmed, we suggest that differences in the degree to which dopamine type 2 and serotonin receptors are blocked by olanzapine and haloperidol (the latter principally blocks dopamine type 2 receptors) underlie the differences in histone modification associated with these two agents. In previous studies, clozapine, olanzapine, and sulpiride, but not haloperidol or risperidone, increased GABAergic promoter demethylation. This suggests that dibenzazepine derivatives (e.g., clozapine and olanzapine) may attenuate the dysregulation of GABAergic and glutamatergic transmission by reducing promoter hypermethylation [71].

Although the present study is the first to investigate the effects of olanzapine on the epigenetic mechanisms involved in BDNF gene transcription in the rat hippocampus following 21 d restraint stress, it is not without limitations. First, the protein levels of BDNF, DNMT1, DNMT3a, and HDAC5 were not directly measured; therefore, further studies measuring these proteins using Western blot analyses are required to strengthen the present findings. Second, the behavioral effects of 21 d restraint stress were not evaluated in the rats. If the epigenetic changes induced by olanzapine can be synchronized with behavioral changes, then the results of the present study may be strengthened. Third, although we examined MeCP2 binding to methylated CpG sites, the levels of DNA methylation in BDNF promoter IV were not measured. Increased MeCP2 occupancy reduces BDNF transcription [54], but it is not known if chronic restraint stress-associated increases in MeCP2 occupancy are associated with the hypomethylation of BDNF promoter IV. We also observed an increase in DNMT1 and DNMT3a expression levels following chronic restraint stress. Additional studies on DNA methylation are needed to determine whether there is a causal relationship between DNA methylation and DNMT expression. Fourth, the dosages of olanzapine and haloperidol used in the present study are not applicable to human subjects. Therefore, future studies with clinical doses of antipsychotics are necessary to determine if they will produce the same beneficial effects on epigenetic regulation of the BDNF gene.

In summary, the present study found that 21 d restraint stress and olanzapine treatment both altered the epigenetic regulation of the BDNF gene in the rat hippocampus. More specifically, 21 d restraint stress affected BDNF mRNA expression levels and the epigenetic regulations of BDNF promoter exon IV, HDAC5, DNMT1, and DNMT3a in the rat hippocampus. However, these changes were reversed following administration of the antipsychotic drug olanzapine. Thus, the present findings suggest that antipsychotic drugs can alter epigenetic mechanisms and that epigenetic regulation may represent an additional mechanism underlying the effects of antipsychotic drugs.

## Figures and Tables

**Table 1:** Individual-level data for each group analyzed by qRT-PCR and ChIP.

	− Restraint stress			+ Restraint stress		
	VEH	OLA	HAL	VEH	OLA	HAL
Total BDNF mRNA	1.00 ± 0.07	1.38 ± 0.05	0.89 ± 0.09	0.54 ± 0.04	1.03 ± 0.10	0.65 ± 0.14
BDNF exon IV mRNA	1.00 ± 0.06	1.55 ± 0.05	0.99 ± 0.07	0.55 ± 0.06	1.10 ± 0.12	0.57 ± 0.26
Acetyl-H3	1.00 ± 0.20	1.44 ± 0.22	1.00 ± 0.37	0.37 ± 0.40	0.88 ± 0.39	0.36 ± 0.29
MeCP2	1.00 ± 0.37	0.64 ± 0.21	1.21 ± 0.27	1.51 ± 0.28	0.73 ± 0.28	1.25 ± 0.22
HDAC5 mRNA	1.00 ± 0.05	0.80 ± 0.06	1.06 ± 0.13	1.47 ± 0.06	0.72 ± 0.14	1.35 ± 0.07
DNMT1 mRNA	1.00 ± 0.09	0.73 ± 0.08	1.05 ± 0.09	1.44 ± 0.11	0.81 ± 0.14	1.20 ± 0.08
DNMT3a mRNA	1.00 ± 0.04	0.64 ± 0.05	1.09 ± 0.09	1.80 ± 0.12	1.21 ± 0.09	1.72 ± 0.13

## Supplementary Materials

Supplementary Materials Figure S1: BDNF expression patterns according to time (2 and 6 h) and duration (1, 7, and 21 d) of restraint stress. Rats (*n* = 6 animals/group) were immobilized for 2 (RS 2 h) or 6 h (RS 6 h) per day over the course of 1, 7, or 21 d. BDNF expression levels in brain homogenates from the hippocampus were detected by SDS-PAGE and Western blot analyses using anti-BDNF antibodies. A representative image and quantitative analysis normalized to the levels of *α*-tubulin are shown. Results are expressed as a percentage of the corresponding data for the control group (CON; no restraint stress) and represent the mean ± standard error of the mean (SEM) of six animals per group. ^∗^ *p* < 0.05 versus control; ^∗∗^ *p* < 0.01 versus control.
