# Supplementary material for: Effects of Antipsychotic Drugs on the Epigenetic Modification of Brain-Derived Neurotrophic Factor Gene Expression in the Hippocampi of Chronic Restraint Stress Rats
Source: Neural Plast. 2018 Jun 11;2018:2682037. doi: 10.1155/2018/2682037 (PMC6016229; doi:10.1155/2018/2682037)
Supplement: Supplementary Materials — Figure S1: BDNF expression patterns according to time (2 and 6 h) and duration (1, 7, and 21 d) of restraint stress. Rats (n = 6 animals/group) were immobilized for 2 (RS 2 h) or 6 h (RS 6 h) per day over the course of 1, 7, or 21 d. BDNF expression levels in brain homogenates from the hippocampus were detected by SDS-PAGE and Western blot analyses using anti-BDNF antibodies. A representative image and quantitative analysis normalized to the levels of α-tubulin are shown. Results are expressed as a percentage of the corresponding data for the control group (CON; no restraint stress) and represent the mean ± standard error of the mean (SEM) of six animals per group. ∗ p < 0.05 versus control; ∗∗ p < 0.01 versus control. [file 2682037.f1.docx]

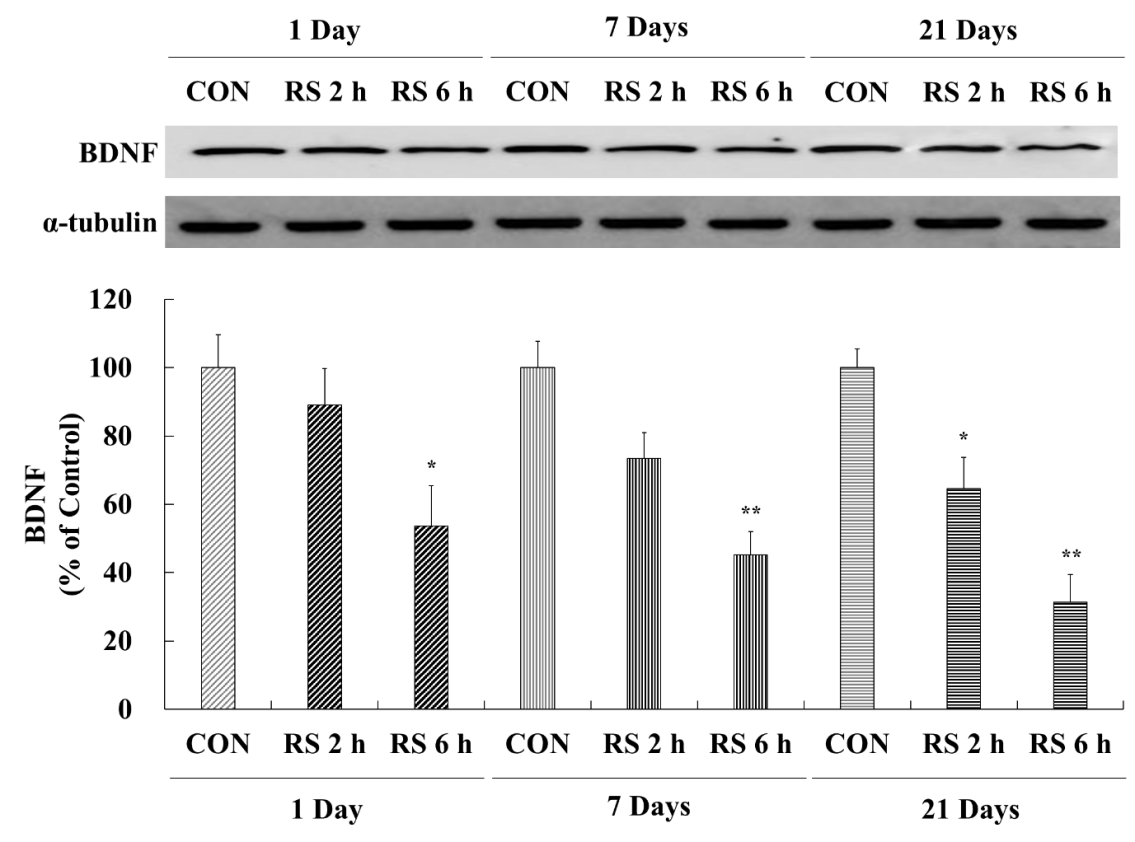


**FIGURE S1: BDNF expression patterns according to time (2 and 6 h) and duration (1, 7, and 21 d) of restraint stress.**

Rats (*n* = 6 animals/group) were immobilized for 2 (RS 2 h) or 6 h (RS 6 h) per day over the course of 1, 7, or 21 d. BDNF expression levels in brain homogenates from the hippocampus were detected by SDS-PAGE and Western blot analyses using anti-BDNF antibodies. A representative image and quantitative analysis normalized to the levels of α-tubulin are shown. Results are expressed as a percentage of the corresponding data for the control group (CON; no restraint stress) and represent the mean ± standard error of the mean (SEM) of six animals per group. ^*^*p* < 0.05 vs. control; ^**^*p* < 0.01 vs. control.
